# Optimisation and Characterisation of Lipase-Catalysed Synthesis of a Kojic Monooleate Ester in a Solvent-Free System by Response Surface Methodology

**DOI:** 10.1371/journal.pone.0144664

**Published:** 2015-12-14

**Authors:** Khairulazhar Jumbri, Mohd Fahruddin Al-Haniff Rozy, Siti Efliza Ashari, Rosfarizan Mohamad, Mahiran Basri, Hamid Reza Fard Masoumi

**Affiliations:** 1 Department of Chemistry, Faculty of Science, Universiti Putra Malaysia, UPM Serdang, Selangor, Malaysia; 2 Department of Technology Bioprocess, Faculty of Biotechnology and Biomolecular Sciences, Universiti Putra Malaysia, UPM Serdang, Selangor, Malaysia; 3 Enzyme and Microbial Technology Research Centre, Universiti Putra Malaysia, UPM Serdang, Selangor, Malaysia; Wageningen University, NETHERLANDS

## Abstract

Kojic acid is widely used to inhibit the browning effect of tyrosinase in cosmetic and food industries. In this work, synthesis of kojic monooleate ester (KMO) was carried out using lipase-catalysed esterification of kojic acid and oleic acid in a solvent-free system. Response Surface Methodology (RSM) based on central composite rotatable design (CCRD) was used to optimise the main important reaction variables, such as enzyme amount, reaction temperature, substrate molar ratio, and reaction time along with immobilised lipase from *Candida Antarctica* (Novozym 435) as a biocatalyst. The RSM data indicated that the reaction temperature was less significant in comparison to other factors for the production of a KMO ester. By using this statistical analysis, a quadratic model was developed in order to correlate the preparation variable to the response (reaction yield). The optimum conditions for the enzymatic synthesis of KMO were as follows: an enzyme amount of 2.0 wt%, reaction temperature of 83.69°C, substrate molar ratio of 1:2.37 (mmole kojic acid:oleic acid) and a reaction time of 300.0 min. Under these conditions, the actual yield percentage obtained was 42.09%, which is comparably well with the maximum predicted value of 44.46%. Under the optimal conditions, Novozym 435 could be reused for 5 cycles for KMO production percentage yield of at least 40%. The results demonstrated that statistical analysis using RSM can be used efficiently to optimise the production of a KMO ester. Moreover, the optimum conditions obtained can be applied to scale-up the process and minimise the cost.

## Introduction

Kojic acid (5-hydroxy-2-hydroxymethyl-4H-pyran-4-one) is an antibiotic produced by many species of *Aspergillus*, *Acetobacter* and *Penicillium* in an aerobic process utilizing a wide range of carbon sources [1]. Kojic acid is extensively used in the food industry as an inhibitor of tyrosinase to prevent browning [2]. Kojic acid can also be used as a skin-lightening or bleaching agent in cosmetic preparations [2,3]. This natural compound is not a very stable compound and when exposed to the sun or air, it will lose its effectiveness as a skin care product. Derivatives of kojic acid are 15 times more stable than the source material, making them more desirable in cosmetic applications in terms of storage stability, compatibility, and oil-solubility [3]. Ariff *et al*. [4] studied the determination of cellular and mushroom tyrosinase activities towards kojic acid esters. The results reported that kojic acid esters were safe and nontoxic depigmenting agents with a satisfactory inhibitory effect on tyrosinase activity as determined in B16F1 melanoma cells. Thus, these compounds have potential to be used in cosmetic formulation. In order to improve the lipophilic properties of kojic acid, modification through esterification with fatty acids is the ideal method to synthesize kojic acid esters [5–7].

Traditionally, optimisation was accomplished through conventional (parameter-at-one-time) methods, which are expensive and time consuming [6]. The now-available multivariate statistic method, including Response Surface Methodology (RSM), is preferred. Response Surface Methodology (RSM) is a collection of mathematical and statistical methods for designing experiments, building models, evaluating the effect of variables, and obtaining the optimum conditions by using a minimal number of experiments [8]. Therefore, the number of trials needed to analyse the interaction between multiple variables can be reduced. RSM was successfully applied to the optimisation of enzymatic syntheses of oleyl oleate and many other esters.

Environmental issues are the main considerations in the synthesis of new chemicals. Previous studies have shown that kojic acid ester can be synthesized using solvent, but this process is not favourable due to low yields. In order to overcome this problem, an enzymatic synthesis of kojic acid ester must satisfy both the demand of production yields while also being an environmentally friendly process. Instead of using an organic solvent media to perform enzymatic reactions, a solvent-free system was studied as an alternative with several advantages. A solvent-free system is a safer process that reduces the extraction cost and increases the reactant concentration and thus volumetric productivity [9]. Most of the research on the production of kojic acid derivatives used organic solvents [7].

**Fig 1:**
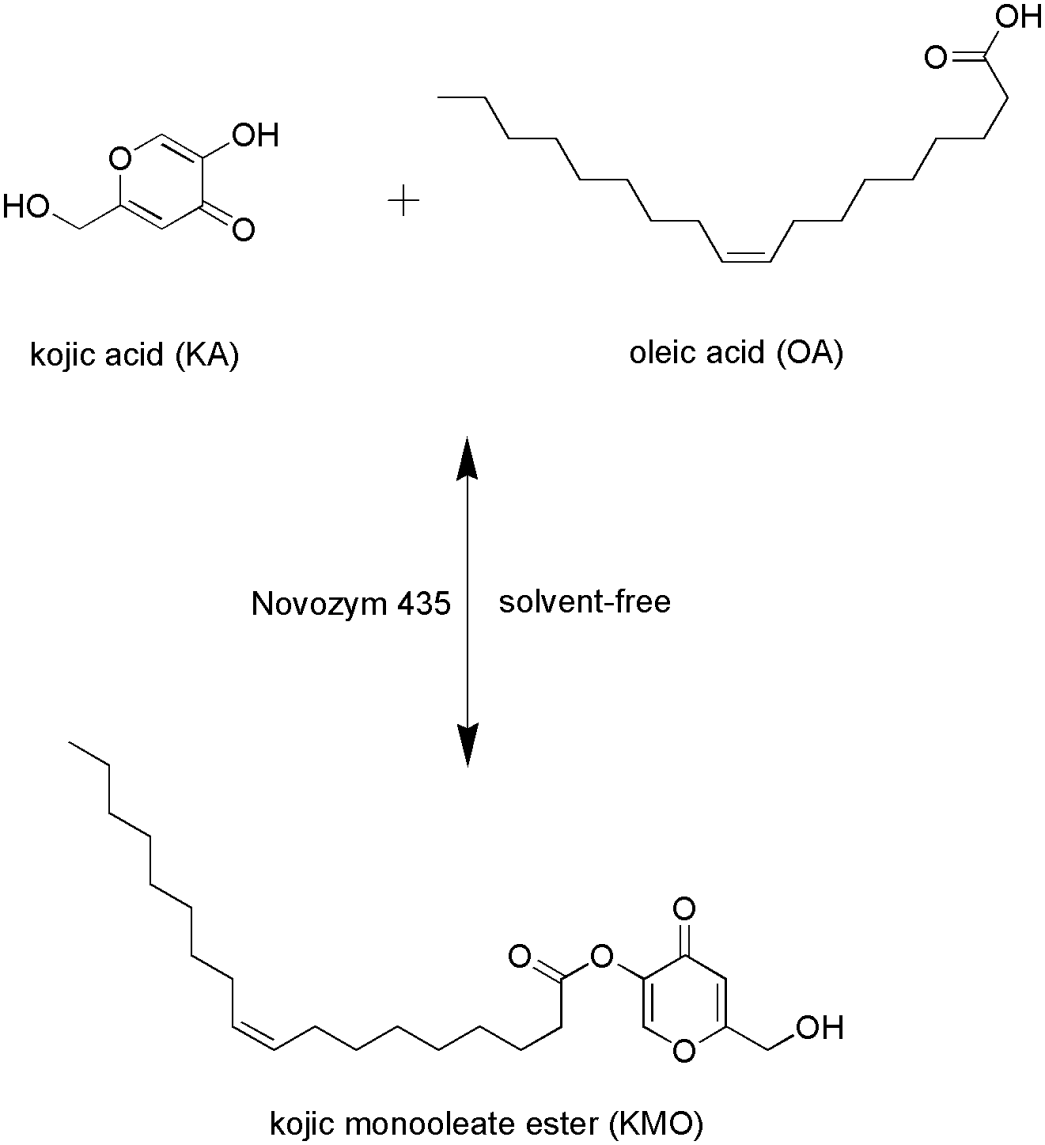
Enzymatic synthesis of kojic monooleate ester in solvent-free system.

The present work focuses on the reaction variables that affect the synthesis of a kojic acid monooleate (KMO) ester catalysed by Novozym 435 in a solvent-free system (Fig 1). The novelty of the reported results is the use of solvent-free system in the enzymatic synthesis of KMO ester and utilizing a response surface methodology (RSM) in optimising the reaction conditions. Nowadays, the solvent-free systems have been identified as a potential alternative for KA ester production and eliminate the problems regarding the use of toxic organic solvents. The objectives of this study were to simultaneously optimise the reaction conditions of KMO ester synthesis in order to attain the best system performance and to understand the relationship between several reaction variables (enzyme amount, reaction temperature, substrate molar ratio, and reaction time) and the response (yield) using RSM.

## Materials and Methods

### Materials

Novozym 435, *Candida Antarctica* lipase B immobilised on a macroporous acrylic resin (10,000 propyl laurate units per gram), was purchased from Novo Nordisk A/S (Denmark). Kojic acid, (purity 98%) was kindly donated by Tokyo Kasei Kogyo Co., Ltd. (Japan). Oleic acid (purity 99%) was obtained for Southern Edible Oil Sdn. Bhd., Selangor, Malaysia. Glycerol tributyrate used as internal standard in gas chromatography analysis was obtained from Sigma-Aldrich (USA). All other chemicals used in this study were of analytical reagent grade.

### Enzymatic esterification and analysis of samples

Kojic monooleate ester (KMO) was synthesized by mixing kojic acid, oleic acid and lipase powder (Novozym 435) in a 150 mL beaker without using any solvent. The beaker containing the mixture was covered using aluminum foil and put into an oil bath. The mixture was stirred using a magnetic stirrer and digital laboratory plate heater with a speed of 240 rpm at 70°C for about 7 hours. At the end of the reaction, the mixture was filtered using filter paper to remove enzyme particles. The mixture was put into the rotary evaporator for a few minutes to make sure that there was no water left in the mixture.

To investigate the reusability of enzyme, after each reaction cycle, Novozym 435 was filtered and washed with hexane for three times to remove any substrate or product. Then, the lipase was dried and consecutively reused in the next reaction.

The effect of added water on the esterification reaction was determined by removing water from organic media by a 3 Å molecular sieves (Merck) at various added water of 0, 10, 20, 30 and 40%. The volume of added water was then calculated by the weight of kojic acid.

The ester formation was then confirmed by thin layer chromatography (TLC) using hexane and ethyl acetate (70:30, v/v) as the developing system. The amount of ester formation was calculated using gas chromatography (GC). Further identification of the ester formation was carried out using gas-chromatography/mass spectroscopy (GC-MS) on a Shimadzu (model GC 17A; model MS QP5050A, Japan) and fourier-transform infrared (FTIR) (Perkin Elmer, model 1650, UK) instruments. ^1^H and ^13^C NMR spectra were recorded for solution of pure KMO ester with tetramethylsilane as an internal standard in CDCl_3_ using a Varian XL-300 spectrometer operating at 30°C and 300 MHz.

### Experimental design and statistical analysis

A central composite rotatable design (CCRD) was employed, which allows for an efficient estimation of the first- and second-order coefficient mathematical model. Many of the previous works used CCRD for optimisation their products [7,10,11]. The generalised response surface model is shown by Eq 1.

$$Y\;=\;\beta_{0}+\;\overset{4}{\underset{i=1}{\sum }}\beta_{i}x_{i}+\;\overset{4}{\underset{i=1}{\sum }}\beta_{ii}x_{i}^{2}+\;\overset{3}{\underset{i=1}{\sum }}\overset{4}{\underset{j=i+1}{\sum }}\beta_{ij}x_{i}x_{j}+\;\varepsilon$$ (1)

where *Y* (yield %) represents the response variable, *β*_0_ is the constant term, *β*_*i*_ represents the coefficients of the linear parameters, *x*_*i*_ represents the variables, *β*_*ii*_ represents the coefficients of the quadratic parameter, *β*_*ij*_ represents the coefficients of the interaction parameters, and *ε* is the residual associated to the experiments.

The reaction undergoes at various reaction temperatures (70–90°C), reaction time (120–360 min), enzyme amount (1.0–5.0 wt%) and substrates molar ratio (1–5 mmole) for kojic acid and oleic acid, which requires 30 experiments. During all the experiments, the agitation speed was fixed at 240 rpm. Table 1 shows the variables in terms of coded and actual values. The range of parameters was selected based on our preliminary studies (data are not shown). For the temperature, as reported in previous work [6], the immobilised enzyme such as Novozym 435 and Lipozyme TL IM are stable and have higher activities at high temperature up to 80°C for 360 min. Furthermore, an increase in the temperature improves the solubility of KA in oleic acid which behaves as a solvent, in solventless reaction, resulting in the enhancement of the reaction rate. This fact can be favourable for the interaction between enzyme particles and the substrates. To ensure accuracy and reproducibility of experiments, repetition experiments were performed. All experiments (30 runs) were carried out in triplicate and standard deviations were less than 5.0%. Consistent results obtained indicating that the experiment was significant with the model based on the R^2^ value.

**Table 1:** Range of variables and their levels for the CCRD.

Variable	Levels				
	–2	–1	0	+1	+2
Reaction temperature, *A* (°C)	70.0	75.0	80.0	85.0	90.0
Reaction time, *B* (min)	120.0	180.0	240.0	300.0	360.0
Enzyme amount, *C* (wt%)	1.0	2.0	3.0	4.0	5.0
Substrates molar ratio, *D* (mole)	1.0	2.0	3.0	4.0	5.0

Each variable was investigated at five levels. An analysis of variance (ANOVA) was performed to determine the significant differences between the independent variables. A reduced model involves statistically independent variables that it takes into account. Multiple regressions were applied in analysing experimental data to predict the coefficients of the fitted second-order polynomial model. Experimental values were compared with the predicted values to check the adequacy of the final reduced model. The recommended optimum conditions were also performed to verify the optimum response values predicted by the model.

## Results and Discussion

### Model fitting and analysis of variance (ANOVA)

In order to obtain a proper model for optimisation of KMO ester synthesis in a solvent-free system, the CCRD, which is the best design for response surface optimisation, was selected with four factors five levels including reaction temperature, reaction time, enzyme amount, and substrate molar ratio. The experimental and predicted yield data are tabulated in Table 2. The predicted values were obtained using a model fitting technique and show sufficiently correlated to the actual values. Fitting of the data to the various models (linear, two factorial interaction, quadratic and cubic) and their subsequent ANOVA shows that the reaction of kojic acid and oleic acid in a solvent-free system is most suitably described with a quadratic polynomial model as shown in Eq 2.

$$Yield\;\left(\%\right)\;=\;+\;29.37\;–\;0.16A\;+3.17B\;–\;3.30C\;+\;2.19D\;–\;7.21AC\;–\;8.58AD\;–\;3.86BC\;–\;4.89B^{2}\;+\;1.87C^{2}\;–\;8.69D^{2}$$ (2)

where *A* is the reaction temperature, *B* is the reaction time, *C* is the enzyme amount, and *D* is the substrates molar ratio. The positive sign in front of the terms indicates a synergistic effect whereas the negative sign indicates an antagonistic effect. Negative values of a coefficient estimate denote a negative influence of parameters on the reaction yield. This quadratic model was found to have coefficient of determination value (*R* ^2^) of 0.9554, which means that 95.54% of the total variation in the observed findings was attributed to the independent variables. Normally, a regression model with the *R* ^2^ value above 0.9 is considered as a model having high correlation [12]. Furthermore, previous studies have stated that the better empirical model fits the actual data can be obtained when *R* ^2^ close to unity value [13]. Therefore, the high value of *R* ^2^ obtained in this regression model indicates a good agreement between predicted and actual yield of KMO ester.

**Table 2:** Central composite rotatable design of KMO ester.

Run no.	Reaction temperature, *A* (°C)	Reaction time, *B* (min)	Enzyme amount, *C* (wt%)	Substrate molar ratio, *D* (mmole)	Yield (%)	
					Actual	Predicted
**1**	**80.0 (0)**	**240.0 (0)**	**3.0 (0)**	**5.0 (+2)**	**1.16**	**1.00**
**2**	**80.0 (0)**	**240.0 (0)**	**3.0 (0)**	**3.0 (0)**	**33.70**	**29.37**
**3**	**75.0 (-1)**	**180.0 (-1)**	**2.0 (-1)**	**4.0 (+1)**	**12.27**	**12.22**
**4**	**80.0 (0)**	**240.0 (0)**	**3.0 (0)**	**3.0 (0)**	**34.95**	**29.37**
**5**	**75.0 (-1)**	**180.0 (-1)**	**2.0 (-1)**	**2.0 (-1)**	**0.25**	**1.54**
**7**	**85.0 (+1)**	**300.0 (+1)**	**4.0 (+1)**	**2.0 (-1)**	**5.43**	**7.28**
**8**	**80.0 (0)**	**360.0 (+2)**	**3.0 (0)**	**3.0 (0)**	**16.62**	**16.15**
**9**	**70.0 (-2)**	**240.0 (0)**	**3.0 (0)**	**3.0 (0)**	**25.75**	**26.69**
**10**	**85.0 (+1)**	**300.0 (+1)**	**2.0 (-1)**	**4.0 (+1)**	**26.03**	**23.22**
**11**	**80.0 (0)**	**240.0 (0)**	**3.0 (0)**	**3.0 (0)**	**30.24**	**29.37**
**12**	**75.0 (-1)**	**300.0 (+1)**	**2.0 (-1)**	**2.0 (-1)**	**17.20**	**15.60**
**14**	**75.0 (-1)**	**180.0 (-1)**	**4.0 (+1)**	**2.0 (-1)**	**5.43**	**6.22**
**15**	**80.0 (0)**	**240.0 (0)**	**3.0 (0)**	**3.0 (0)**	**30.82**	**29.37**
**17**	**85.0 (+1)**	**180.0 (-1)**	**4.0 (+1)**	**4.0 (+1)**	**3.93**	**6.73**
**18**	**75.0 (-1)**	**300.0 (+1)**	**4.0 (+1)**	**2.0 (-1)**	**5.57**	**4.84**
**20**	**85.0 (+1)**	**180.0 (-1)**	**2.0 (-1)**	**2.0 (-1)**	**35.42**	**32.80**
**21**	**80.0 (0)**	**120.0 (-2)**	**3.0 (0)**	**3.0 (0)**	**5.15**	**3.47**
**22**	**85.0 (+1)**	**180.0 (-1)**	**4.0 (+1)**	**2.0 (-1)**	**10.26**	**8.66**
**23**	**75.0 (-1)**	**300.0 (+1)**	**2.0 (-1)**	**4.0 (+1)**	**24.32**	**26.28**
**24**	**90.0 (+2)**	**240.0 (0)**	**3.0 (0)**	**3.0 (0)**	**27.76**	**29.05**
**25**	**80.0 (0)**	**240.0 (0)**	**5.0 (+2)**	**3.0 (0)**	**30.58**	**30.27**
**26**	**85.0 (+1)**	**180.0 (-1)**	**2.0 (-1)**	**4.0 (+1)**	**4.25**	**9.16**
**27**	**80.0 (0)**	**240.0 (0)**	**3.0 (0)**	**3.0 (0)**	**22.48**	**29.37**
**28**	**85.0 (+1)**	**300.0 (+1)**	**4.0 (+1)**	**4.0 (+1)**	**5.69**	**5.35**
**29**	**80.0 (0)**	**240.0 (0)**	**1.0 (-2)**	**3.0 (0)**	**45.31**	**43.46**
**30**	**80.0 (0)**	**240.0 (0)**	**3.0 (0)**	**3.0 (0)**	**31.41**	**29.37**

**Fig 2:**
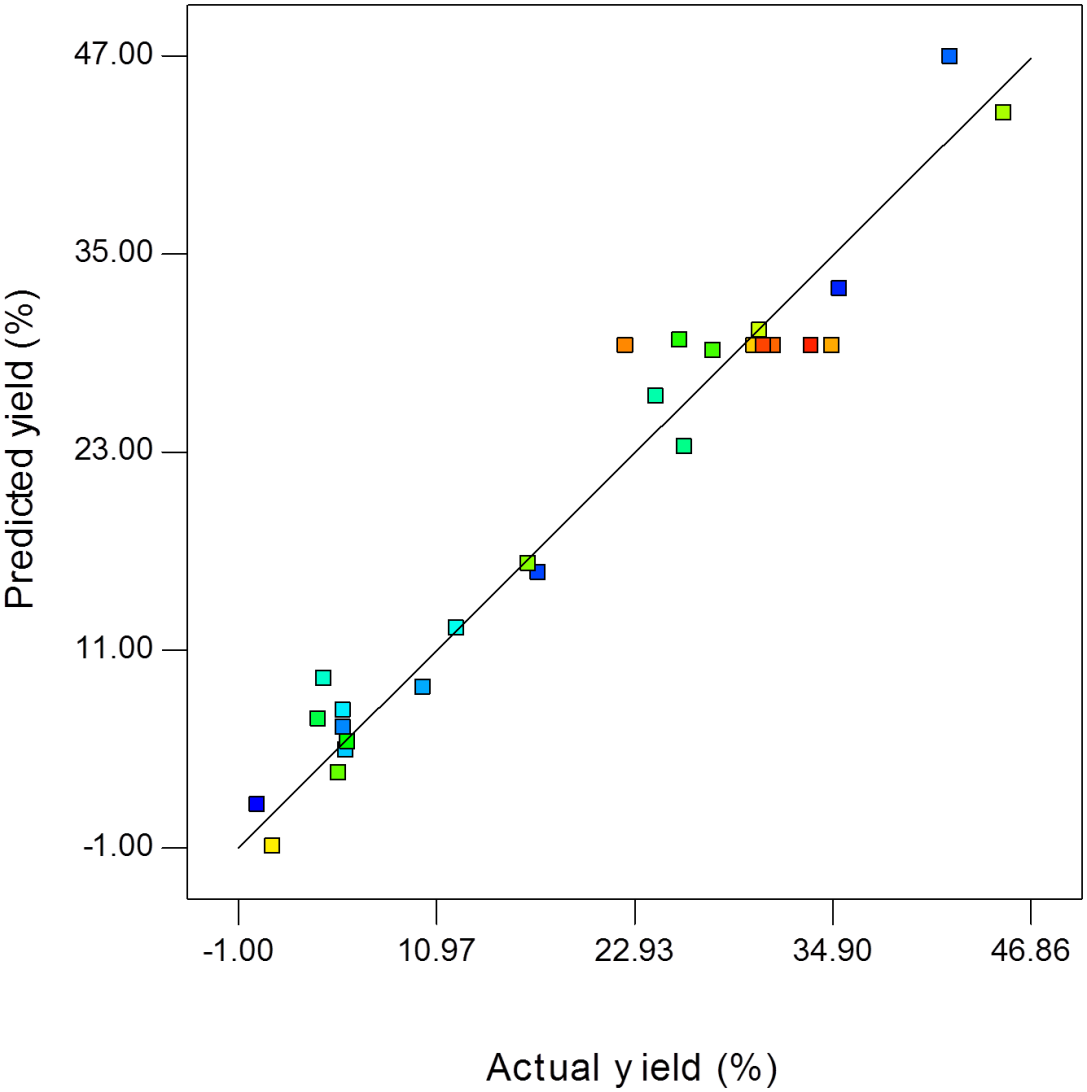
Correlation of actual and predicted values of yield by the response surface model.

The ANOVA for the model is tabulated in Table 3. The computed *F*-value of the model (29.20) implies that the model is significant and the lack-of-fit *F*-value of 0.64 shows that lack-of-fit is not significant relative to pure error. The model of *F*-value was identified as significant as the *P*-value (< 0.0001) was less than 0.05 and there was only 0.01% chance that a “model *F*-value” this large could have occurred due to noise in the experiments. According to Bezerra *et al*. [14], significant regression and a non-significant lack-of-fit present in the model was well fitted to the experiments. Fig 2 shows good correlation between the actual and predicted yield for KMO ester synthesis using Novozym 435 in a solvent-free system. The linear distribution is indicative of a well-fitted model. The generated models were employed subsequently to study the effect of various parameters and their interactions on the yield of reaction.

**Table 3:** ANOVA for the quadratic model developed for synthesis of KMO ester.

Source	Sum of Squares	Degree of Freedom	Mean Square	*F*-value	*P*-value
**Model**	**4660.42**	**11**	**423.67**	**29.20**	**<0.0001**
**Reaction temperature, *A***	**0.47**	**1**	**0.47**	**0.03**	**0.8600**
**Reaction time, *B***	**218.20**	**1**	**218.20**	**15.04**	**0.0015**
**Enzyme amount, *C***	**202.79**	**1**	**202.79**	**13.98**	**0.0020**
**Substrate molar ratio, *D***	**64.72**	**1**	**64.72**	**4.46**	**0.0519**
**AC**	**581.77**	**1**	**581.77**	**40.10**	**< 0.0001**
**AD**	**824.93**	**1**	**824.93**	**56.86**	**< 0.0001**
**BC**	**205.89**	**1**	**205.89**	**14.19**	**0.0019**
**CD**	**330.35**	**1**	**330.35**	**22.77**	**0.0002**
**B** ^**2**^	**642.97**	**1**	**642.97**	**44.32**	**< 0.0001**
**C** ^**2**^	**94.42**	**1**	**94.42**	**6.51**	**0.0221**
**D** ^**2**^	**1207.33**	**1**	**1207.33**	**83.22**	**< 0.0001**
**Residual**	**217.61**	**15**	**14.51**	**-**	**-**
**Lack-of-fit**	**122.31**	**10**	**12.23**	**0.64**	**0.7429**
**Pure error**	**95.30**	**5**	**19.06**	**-**	**-**
**Corrected total**	**4878.03**	**26**	**-**	**-**	**-**

### Effect of Reaction Parameters

The relationship between reaction parameters and responses can be understood by studying the three-dimensional (3D) response surface plots generated from the predicted quadratic model. The 3D response surface plot can also be used to determine the optimum level of each variable for production of KMO ester. While maintaining other variables at their optimal level, Z-axis (refer to percentage of yield) against any two variables was constructed in response surface plot.

**Fig 3:**
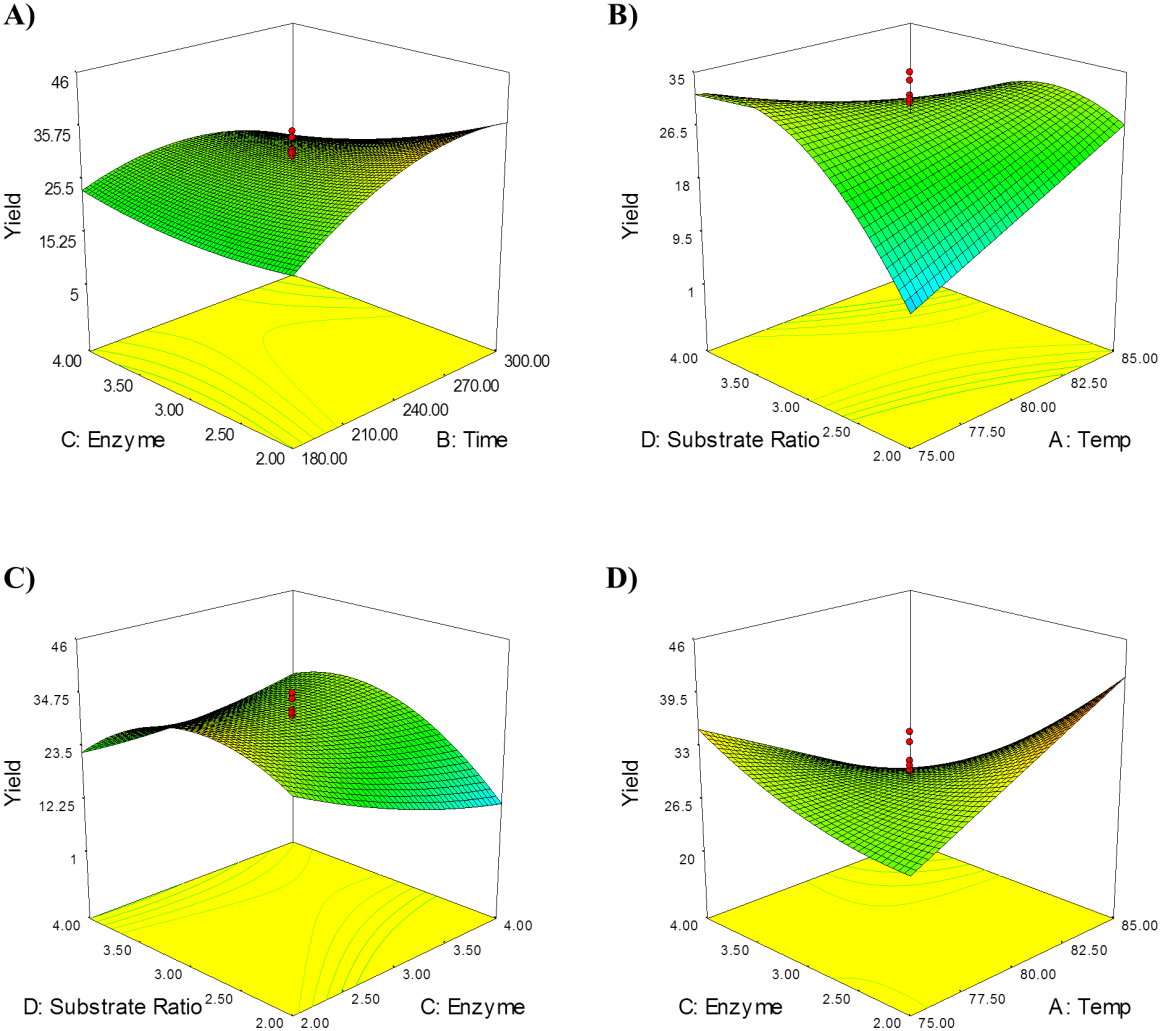
Three-dimensional response surface plots. (A) enzyme amount (%wt) *versus* reaction time (min), (B) substrate molar ratio (mmole) *versus* temperature (°C), (C) enzyme amount (%wt) *versus* substrate ratio (mmole), and (D) enzyme amount (%wt) *versus* reaction temperature (°C) on percentage yield as response.

As shown in Fig 3A, amount of enzyme and the reaction time were interpreted in the range of 2.0–4.0 wt% and 180–300 min, respectively with the reaction temperature fixed at 80°C and 1:3 substrates molar ratio (KA:OA). At the beginning, it was observed that the increase of reaction time increases the percentage of yield and the highest yield (35.75%) was achieved with 276.0 min and 2.0 wt% of enzyme. Therefore, this result indicated that prolonging the reaction time (>276 min) will not increase the production of KMO because this esterification reaction produced water as aside product. When the reaction time increases, the concentration of the product also increases, therefore more water can be produced and this situation may promote reverse esterification [15,16]. Increasing the enzyme amount would not contribute in obtaining the conversion of KMO because steric hindrance could be produced by an excessive enzyme [17].

Fig 3B represents the effect of reaction temperature and substrates molar ratio. Response surface plot was generated with a reaction time fixed at 240 min and 3.0 wt% of enzyme amount for obtaining the interaction of varying reaction temperature (75–85°C) and the substrates molar ratio of KA:OA (1:2–1:4 mmole). The result showed that the highest yield (33.87%) of KMO was obtained at 75°C and a ratio of 1:3.5 (KA:OA). It is true that higher temperature can produce a low conversion as it tended to induce enzyme inactivation due to denaturation processes [18–20]. The lowest percentage of yield was obtained at ratio of 1:2 mmole (KA:OA). As mentioned earlier, this enzymatic reaction was carried out in a solvent-free condition, so in this case oleic acid can also act as a solvent. Lesser than this amount of oleic acid can make the heat transfer and mixing become difficult. Therefore, sufficient amounts of kojic acid was not completely used as the mixture was not fully stirred using magnetic stirrer.

Fig 3C illustrated the effect of varying the amount of enzyme and molar ratio of substrates on the esterification reaction of kojic acid and oleic acid while reaction temperature and reaction time were fixed at 80°C for 240.0 min. It was shown that the maximum conversion of KMO was obtained when 2.0 wt% of enzyme was used to react with 1:3 mmole (KA:OA). In addition, large excesses of enzyme also led to a lower conversion because enzyme molecules can associate with each other, masking the active site that cannot accommodate the substrates, and therefore reducing the yield of the product [21].

Fig 3D represents the effect of varying reaction temperature (75–85°C) and enzyme amount (2.0–4.0 wt%) on the synthesis of KMO. This response surface was plotted at a fixed reaction time (240 min) and fixed substrates molar ratio (1:3). The highest percentage of yield was obtained at 85°C using 2.00 wt% of enzyme whereas the lowest yield was obtained using 85°C with 4.0 wt% of enzyme amount. As mentioned previously, the enzyme amount does affect the percentage of yield because a large amount of enzyme can contribute to a large excess of enzyme active site and it would not be exposed to the substrates due to the possible protein aggregation. The response surface plot patterns show that the increase in reaction temperature can contribute in lowering the conversion of KMO. This probably occurs due to the inactivation of the enzyme at high temperature.

### Optimum Conditions

The response surface can indicate the optimal combination of parameters to obtain the highest percentage yield. As shown previously (Table 2), the highest actual yield obtained was 45.31% using a temperature of 80°C, reaction time of 240 min, amount of enzyme at 1 wt%, and 1:3 substrate molar ratio. The expected maximum yield of 44.46% at reaction conditions of 83.69°C, 300 min, 2.0 wt% of enzyme, and substrate molar ratio of 1:2.37 was predicted by using the optimise function of the Design Expert software. As shown in Table 4, the actual experimental value obtained was 42.09%, with a small difference of 2.37. Therefore, this result confirms the validity of the quadratic model and demonstrated that the response surface methodology can be applied effectively to optimise the lipase-catalysed synthesis of KMO ester.

**Table 4:** Optimum conditions for Novozym 435 catalysed synthesis of KMO ester.

Run no.	Reaction temperature, *A* (°C)	Reaction time, *B* (min)	Enzyme amount, *C* (wt%)	Substrate molar ratio, *D* (mmole)	Yield (%)		Difference
					Actual	Predicted	
1.	83.69	300	2.0	2.37	42.09	44.46	2.37

### Reusability of Enzyme

**Fig 4:**
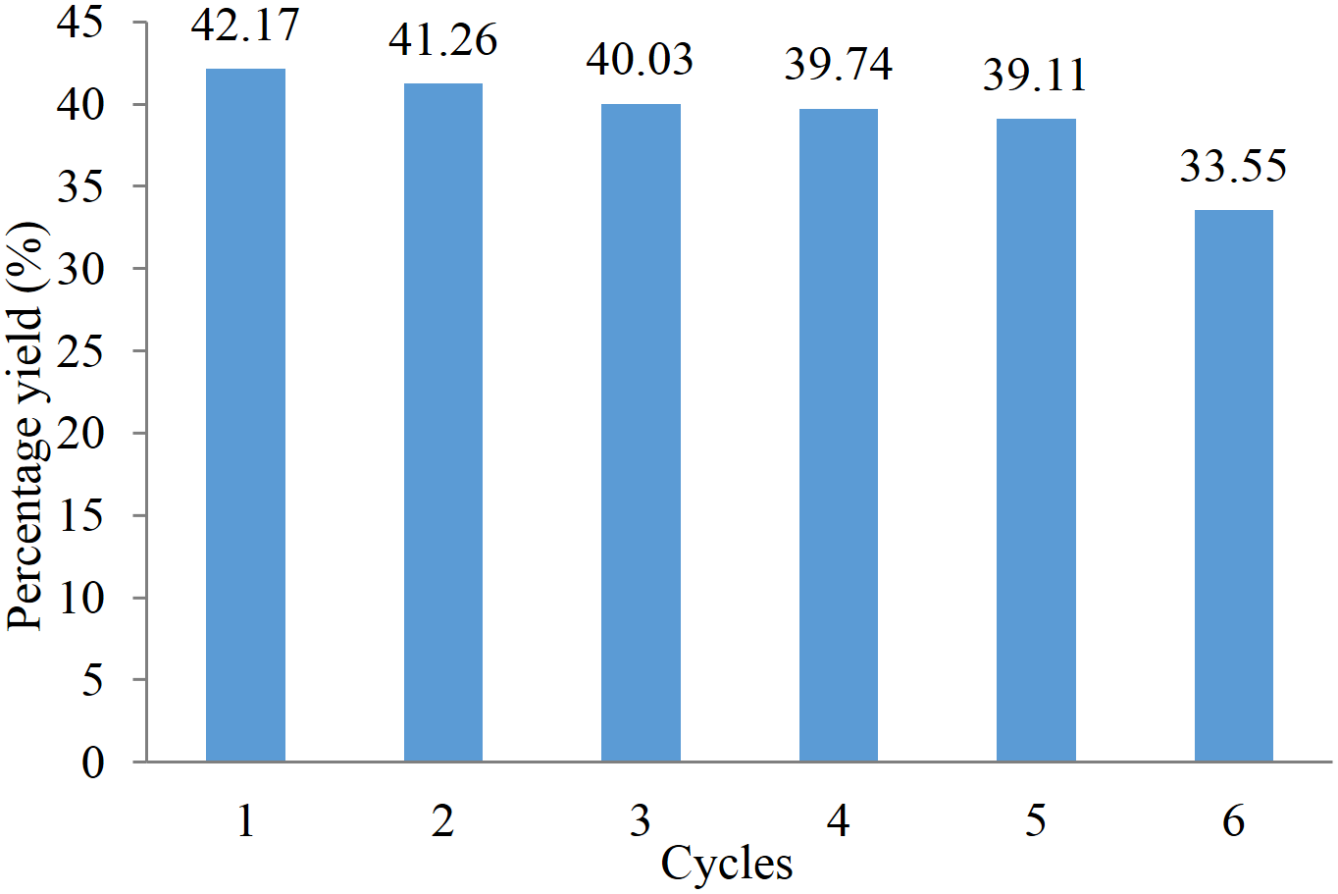
Reusability study of Novozym 435.

The reusability test under the optimised conditions of immobilised lipase in esterification reaction has been studied as shown in Fig 4. It was observed that after 5 cycles, the percentage yield of KMO ester was still acceptable.

### Effect of added water

**Fig 5:**
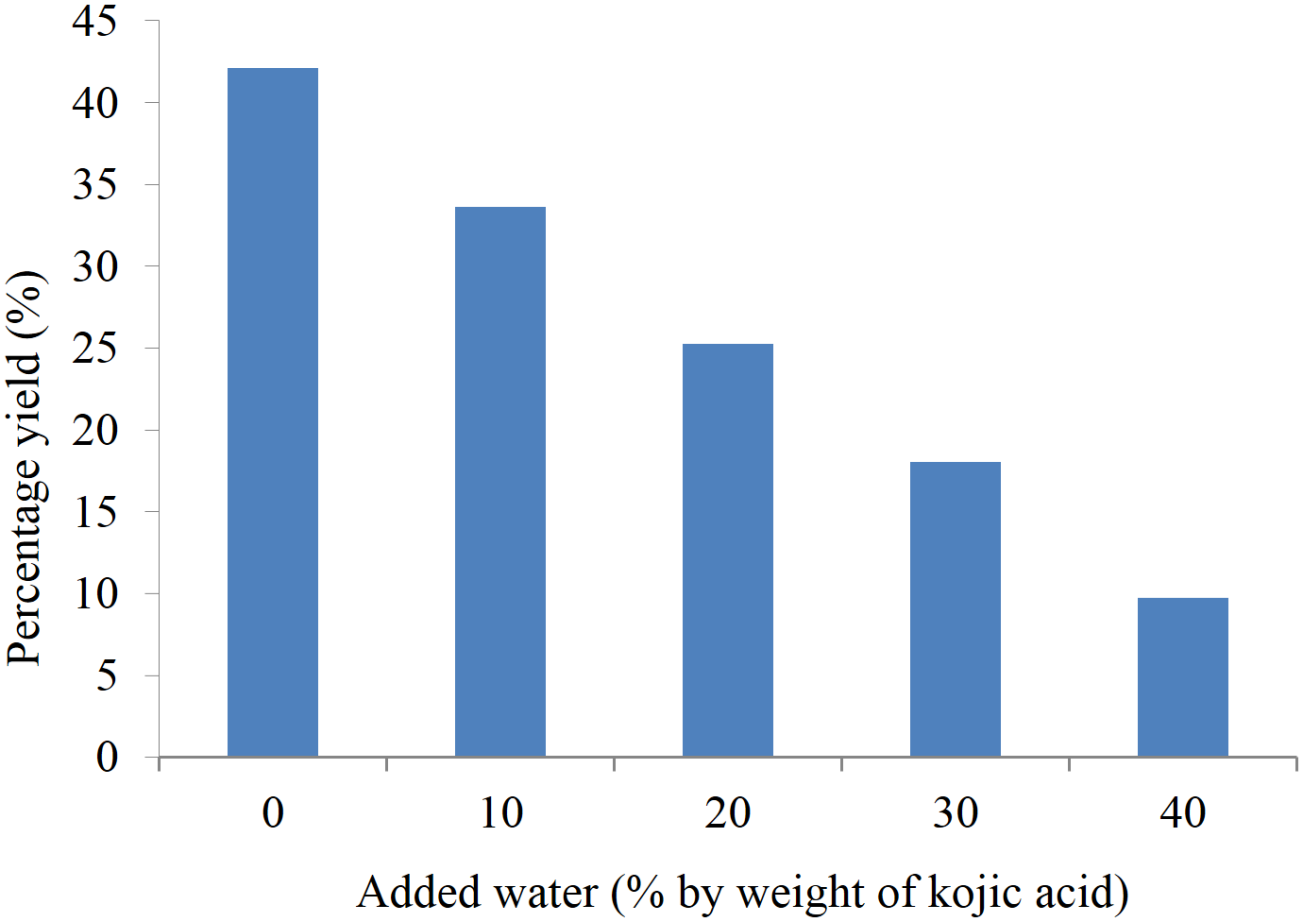
Effect of added water on the KMO synthesis.

Water is necessary for the optimal tertiary conformation of the enzyme. However, too much of water produces negative effects on stability and activity of the enzyme. Fig 5 shows the effect of added water on the synthesis of KMO at various amounts of added water from 0 to 40%. From the observation, at low water content, the percentage yield increased up to 42%. This result confirmed that hydration favoured enzyme activation. At high amount of water, the percentage yield decreased to a minimum at 10%. This is due to the enzyme tended to adsorb to form compact agglomerates. This trend was similar to the work that has been reported in literature [22–23]. They found that added water content in the reaction remarkably lowered the catalytic activity.

### Identification of the Product

**Fig 6:**
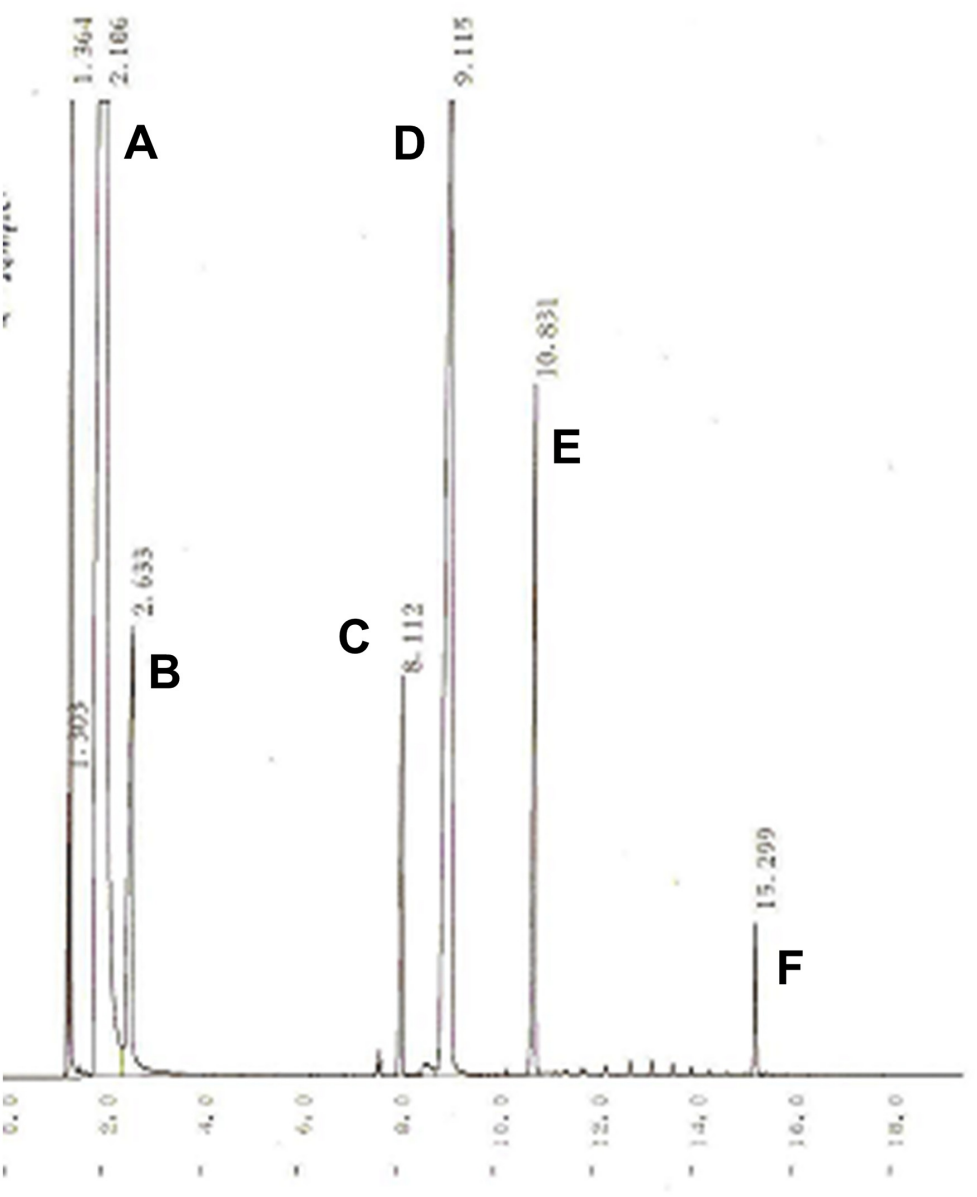
Gas chromatography of palm-based kojic monooleate ester. Peaks A = *N*,*N*-dimethylformamide, B = *N*,*O*-bis-trimethylsilyl acetamide, C = Kojic acid, D = internal standard (1,2,3-tributyrylglycerol), E = oleic acid, F = palm-based kojic monooleate ester (KMO).

The identification of the product was confirmed by TLC, GC, FT-IR, MS and NMR. The chromatogram of the KMO product detected by TLC (R_f_ = 0.27) showed a retention time of 15.299 min when analysed by gas chromatography (GC) as illustrated in Fig 6. The formation of ester and disappearance of substrate monitored by FT-IR showed a medium band at around 3323 cm^-1^ corresponding to the characteristic of hydrogen-bonded O-H vibration. The spectrum also shows the characteristic absorption bands at 2929 and 2894 cm^-1^, which correspond to CH_3_ and CH_2_ stretching, respectively. The strong absorption peak around 1750 to 1730 cm^-1^ indicated the presence of the carboxyl group (C = O). However, the C = O stretching for the expected ester carbonyl gave slightly decreases which an absorption peak at around 1728 cm^-1^ due to the presence of conjugation of C = O with phenyl (kojic acid) which the range is basically decrease to 1740 to 1715 cm^-1^. The mass spectrum shows a molecular ion peak at m/z 478.

^1^H and ^13^C NMR spectrum of KMO are shown in S1 and S2 Figs. The results indicated that the primary hydroxyl group at the C-7 of kojic acid was regioselectivity esterified to afford 7-O-oleate kojic acid. However, this finding is not consistent with Liu and Shaw [5] where they found that the hydroxyl group at the C-5 position of kojic acid was esterified with lauric acid, as well as oleic acid for production of kojic acid monolaurate and kojic acid monooleate using lipase from *Candida antartica* and *P*.*cepacia* in organic solvent. Moreover, the hydroxyl group at the C-5 position of kojic acid can also esterified when a chemical catalyst is used [24]. In order to confirm which hydroxyl group esterified, S3 Fig shows the two-dimensional COSY spectrum which to determine the connectivity of kojic acid and palm-based oleic acid structure by determining which protons and carbons is spin-spin coupled. From the spectrum, it can be seen that the hydroxyl-group at C-7 of kojic acid was easily attached to the palm-based oleic acid to produce KMO. ^1^H NMR (CDCl_3_, TMS): 0.80 (3H, t, *J* = 7.0 Hz, CH_3_), 1.21 (22H, m, (CH_2_)_11_), 1.58 (2H, t, *J* = 7.0 Hz, CH_2_), 1.93 to 1.98 (4H, m, CH_2_CH = CHCH_2_), 2.33 (2H, t, *J* = 7.0 Hz, CH_2_COO), 4.86 (2H, s, CH_2_O), 5.25 to 5.29 (1H, m, CH = CH), 6.44 (1H, s, H3), 7.80 (1H, s, H6). ^13^C NMR: 14.1, 33.5–20.6, 61.1, 111.4, 138.4, 146.1, 162.3, 172.7,174.2.

## Conclusions

In this study, the optimisation of lipase-catalysed esterification of kojic monooleate ester (KMO) in solvent-free system was successfully performed using response surface methodology (RSM). The R^2^ value of 0.9554 indicated a good fit of the model with experimental finding. The ANOVA implied that the model satisfactorily represented the real relationship of the four main reaction variables and the response. A yield of 42.09% was obtained at the optimum conditions that can be used for future up scaling of the process.

## Supporting Information

S1 Fig ^1^H NMR spectrum of palm-based kojic monooleate (KMO). (DOCX)

S2 Fig ^13^C NMR spectrum of palm-based kojic monooleate (KMO). (DOCX)

S3 Fig COSY spectrum of palm-based kojic monooleate (KMO). (DOCX)
