# Supplementary figures and images for: Optimisation and Characterisation of Lipase-Catalysed Synthesis of a Kojic Monooleate Ester in a Solvent-Free System by Response Surface Methodology
Source: PLoS One. 2015 Dec 14;10(12):e0144664. doi: 10.1371/journal.pone.0144664 (PMC4681531; doi:10.1371/journal.pone.0144664)

**
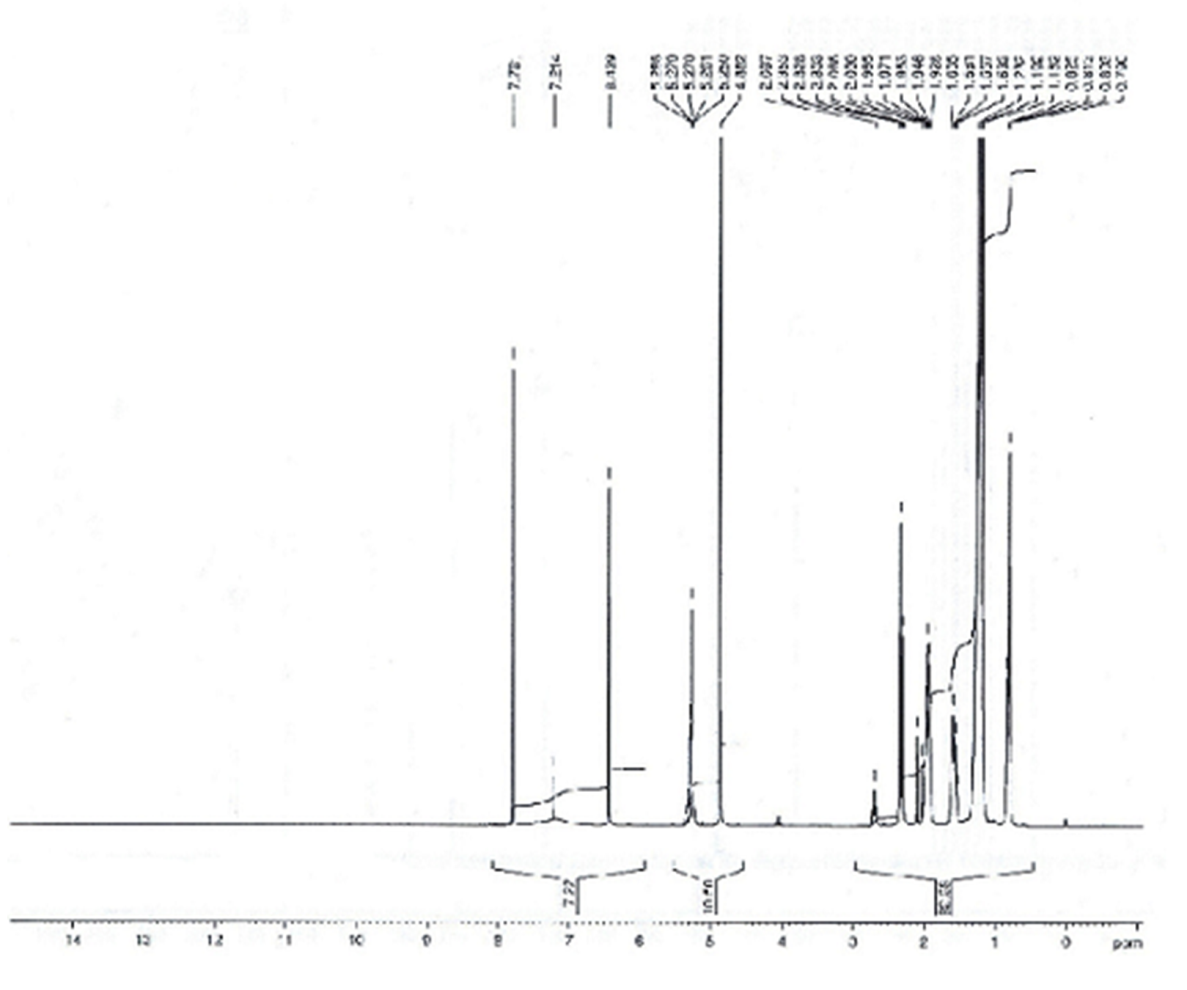
**

**S1 Fig. 1H NMR spectrum of palm-based kojic monooleate (KMO).**

Supplement: S1 Fig — (DOCX) [file pone.0144664.s001.docx]

**
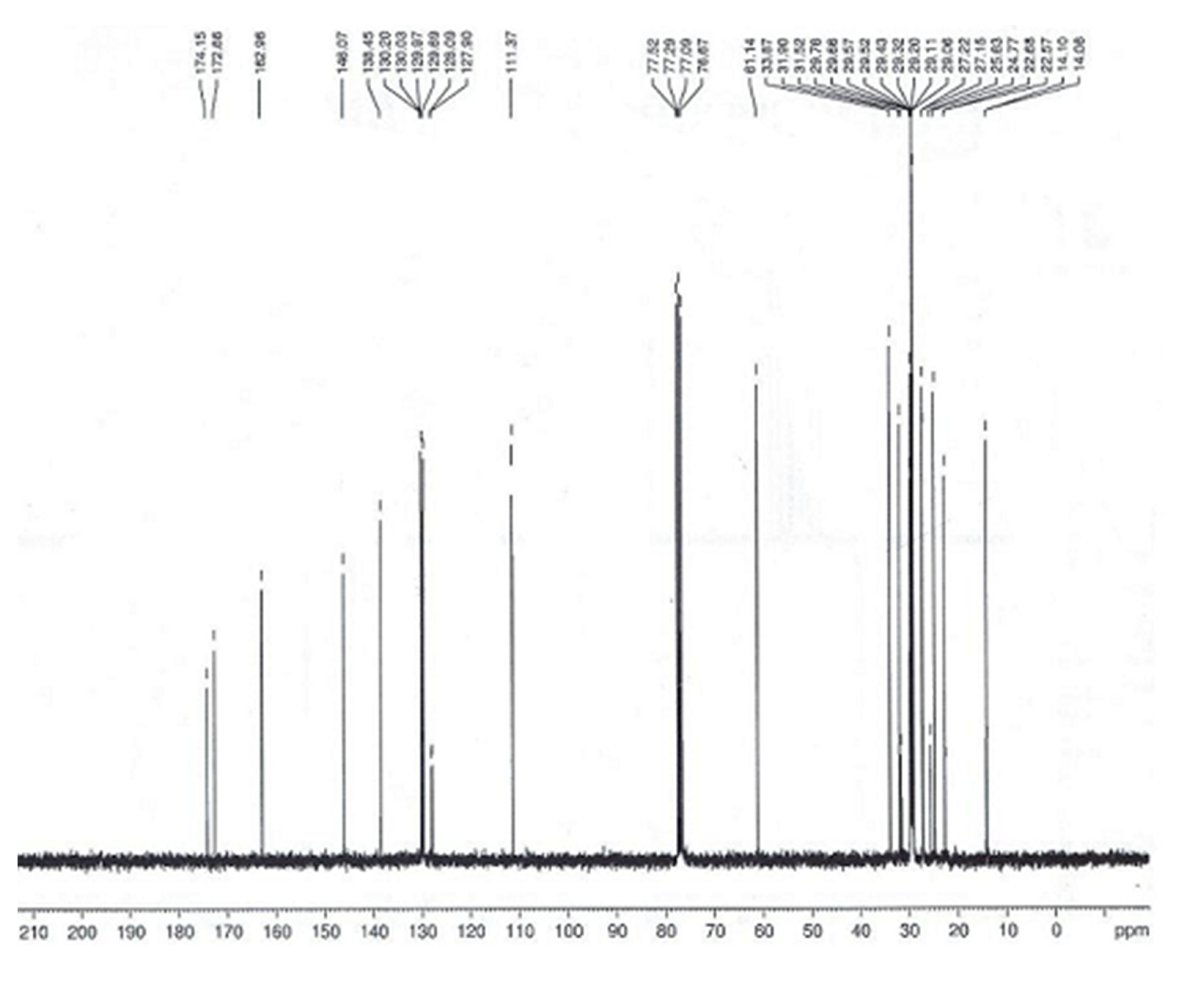
**

**S2 Fig. 13C NMR spectrum of palm-based kojic monooleate (KMO).**

Supplement: S2 Fig — (DOCX) [file pone.0144664.s002.docx]

**
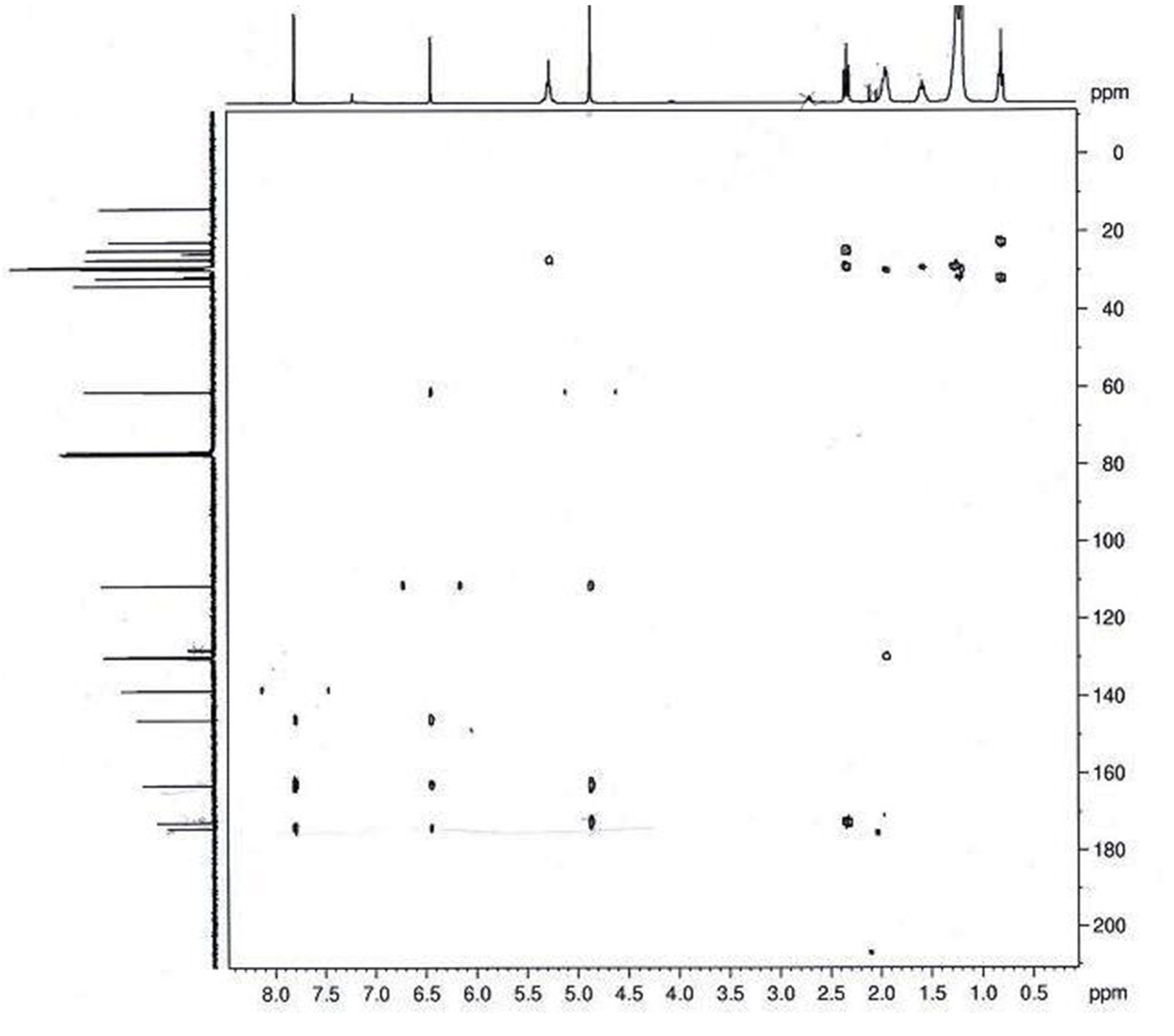
**

**S3 Fig. COSY spectrum of palm-based kojic monooleate (KMO).**

Supplement: S3 Fig — (DOCX) [file pone.0144664.s003.docx]
